# Impacts and Identification of Hearing Aid Refurbishing Programs for People with Hearing Loss: A Scoping Review

**DOI:** 10.3390/audiolres13030028

**Published:** 2023-05-06

**Authors:** Mathieu Hotton, Virginie Prud’Homme, Léa Richard, Laurie Cormier, Katherine Simoneau, Mathilde Lefebvre-Demers, Claude Vincent, Normand Boucher

**Affiliations:** 1 Centre Interdisciplinaire de Recherche en Réadaptation et Intégration Sociale (Cirris), Quebec, QC G1M 2S8, Canada; 2 Rehabilitation Department, Université Laval, Quebec, QC G1V 0A6, Canada; 3 Centre Intégré Universitaire de Santé et de Services Sociaux de la Capitale-Nationale, Quebec, QC G1V 0A6, Canada

**Keywords:** hearing loss, hearing aids, refurbishing, low-income population, rehabilitation, audiology, scoping review

## Abstract

This article consists of a scoping review completed to describe the impacts of refurbished hearing aids (HAs) for people with hearing loss, and to identify existing HA refurbishing programs around the world. In this review, JBI methodological guidance for scoping reviews was followed. All types of sources of evidence were considered. Thirty-six sources of evidence were included, 11 articles and 25 web pages. Results suggest that refurbished HAs may improve communication and social participation for individuals with hearing loss and provide monetary savings to them and to governmental agencies. Twenty-five HA refurbishing programs were identified, all based in developed countries and distributing refurbished HAs mostly locally, but also in developing countries. Issues related to refurbished HAs were highlighted, such as potential cross-contamination, quick obsolescence, and repairing problems. Some facilitators for the success of this intervention are to offer accessible and affordable follow-up services, repairs, and batteries, and to ensure awareness and participation of hearing healthcare professionals and citizens with hearing loss. In conclusion, the use of refurbished HAs appears to be a valuable option for low-income people with hearing loss, but it should be included in a more global intervention program to ensure its sustainability.

## 1. Introduction

Hearing loss is one of the most common chronic health conditions in the world. According to the World Health Organization (WHO), around 1.5 billion people currently live with it around the world, and it is estimated that this number will be close to 2.5 billion by 2050 [1]. The consequences of hearing loss are important and numerous. It can lead to problems with hearing perception and communication, but also to fatigue, anxiety, social isolation, psychological distress, and depression [2,3,4]. Hearing loss is also associated with cognitive decline and a higher risk of falls in the elderly [5]. It can also have repercussions on significant others, who may experience frustration due to the communication difficulties caused by hearing loss and experience an increased feeling of burden associated with the support role they must assume [6,7].

Hearing aids (HAs) are the main assistance technology recommended to improve listening and communication for people with hearing loss. Despite their effectiveness to alleviate activity limitations and participation restrictions experienced by those people and to improve quality of life [8,9], only a small proportion of those people own HAs. Of the estimated 466 million people worldwide, who are candidates for hearing amplification, the number of which includes individuals with moderate to profound hearing loss, only 17% own HAs [1]. Available data suggest that this is also true in developed countries, where the proportion of people with hearing loss owning HAs ranges from 34 to 41% [10,11,12]. Therefore, there is a substantial gap between the need for HAs and the number of people who have access to them.

Many reasons may explain this discrepancy between HA needs and ownership. Among others are the importance of self-perceived hearing difficulties [10], the stigma associated with the use of HAs [11], and the high cost of acquiring and maintaining this technology. In relation to the latter, between 42 and 74% of non-owner respondents to HA surveys mentioned that HAs are too expensive or that they cannot afford them [12,13,14]. To improve access to HAs for people with hearing loss who are in that situation, non-profit community organizations operate HA refurbishing programs, which consist of a bundle of services aiming at recycling used HAs and give them a second life. These organizations collect HAs that are damaged or no longer used (for example, because the owner changed his old HAs for new ones, discontinued the use of HAs, or died). After fixing these HAs as well as cleaning and checking their functionality, organizations donate them to low-income people during humanitarian missions in developing countries or in local communities. In the Province of Québec, in Canada, the Association des Personnes avec une Déficience de l’Audition (APDA) is one of the few organizations offering those services [15]. Informal conversations with the APDA personnel and clinicians around the province (i.e., audiologists and hearing aid specialists) suggest that community-based HA refurbishing programs remain little known from the public and from the hearing healthcare practitioners, which do not refer many clients to those programs. To our knowledge, no data are available about how common HA refurbishment is and the number of people using this type of device in the world. It is known that refurbished HAs circulate in many countries but there are no available data about refurbished HA sales and distribution [16].

This article presents the results of an exploratory scoping review realized to describe the impacts of HA-refurbishing programs for people with hearing loss, and to identify existing HA refurbishing programs around the world. If the impacts of those programs were mostly positive, and if those programs were better known to the public and hearing healthcare professionals, more people in need would be able to gain access to HAs and to benefit from them, which would improve their social participation and quality of life. Three research questions were explored in this scoping review: (1) What are the advantages and disadvantages of refurbished HAs? (2) What are the determining factors for the success of a HA refurbishing program? (3) What are the existing programs which distribute refurbished HAs to people with hearing loss around the world?

## 2. Materials and Methods

### 2.1. Design

A scoping review was conducted [17], following the guidelines by Peters et al. [18]. We used the Preferred Reporting Items for Systematic reviews and Meta-Analysis (PRISMA) guideline extension for scoping reviews (PRISMA-ScR) to guide the reporting of our results [19]. A scoping review protocol was developed and pre-tested by the research team prior to undergoing the review process, but this protocol was not published.

### 2.2. Eligibility Criteria

To answer research questions #1 and #2, the following sources of evidence were considered: quantitative, qualitative, and mixed methods research, intervention and descriptive studies, and reviews. To answer research question #3, all sources of evidence were accepted. To be included, sources of evidence had to specifically address the topic of HA refurbishment for participants with hearing loss of all ages, and to present information useful in answer to one of the two research questions. Documents published in English or French in peer-reviewed scientific or professional journals were considered, along with book chapters, conference abstracts, academic theses, web sites, and policy documents.

### 2.3. Search Strategy

The following databases were consulted: Medline, Embase, CINAHL, PsycINFO, and Web of Science. Each of these data sources were searched using free keywords, combined as follows: [(hearing aid) OR (hearing device) OR (hearing technology) OR (hearing assistance technology) OR (hearing assistive device) OR (hearing assistive aid) OR (assistive listening device) OR (hearing instrument)] AND [(recycl*) OR (refurbish*) OR (valoriz*) OR (valoris*)]. This search strategy is also presented in Table 1. Google Scholar was also consulted. Two text searches were carried out on that search engine (“hearing aid recycl” and “hearing aid refurbish”). No standardized vocabulary (i.e., MeSH Terms), no quotes, and no limits were used to allow databases’ searches to be the broader possible. Reference lists of included sources of evidence were also searched, and contacts were made with key informants to identify other published or unpublished sources that might not have been highlighted by conventional databases’ consultation.

### 2.4. Procedures

Titles of sources of evidence identified with the literature search were reviewed to exclude those that did not meet inclusion criteria. Then, a second validation of inclusion criteria was realized by reviewing the abstracts of the selected articles. This led to a final list of sources of evidence that were included in the complete review process. These sources were read to collect the data pertaining to the research questions. The literature search, the selection of the sources of evidence to be included in the review, and the validation of inclusion criteria were performed independently by two reviewers (VP and LR). At the end of the process, the results of the literature search and the list of studies included by the reviewers were validated by the first author (MH), which led to the final list of included sources of evidence.

### 2.5. Data Extraction

The data extraction was completed independently by two reviewers (VP and LR). Those reviewers did not use a specific extraction form or checklist for completing this task. They essentially summarized the main results of the included sources of evidence in the form of a narrative literature review (for included articles related to research questions #1 and #2) and of a written description of the identified HA refurbishing programs (in relation to research question #3). Those HA refurbishing programs are also presented in a summary table. When the data extraction was completed by the reviewers, the results were reviewed and validated by the first author (MH).

## 3. Results

### 3.1. Included Sources of Evidence

**Figure 1:**
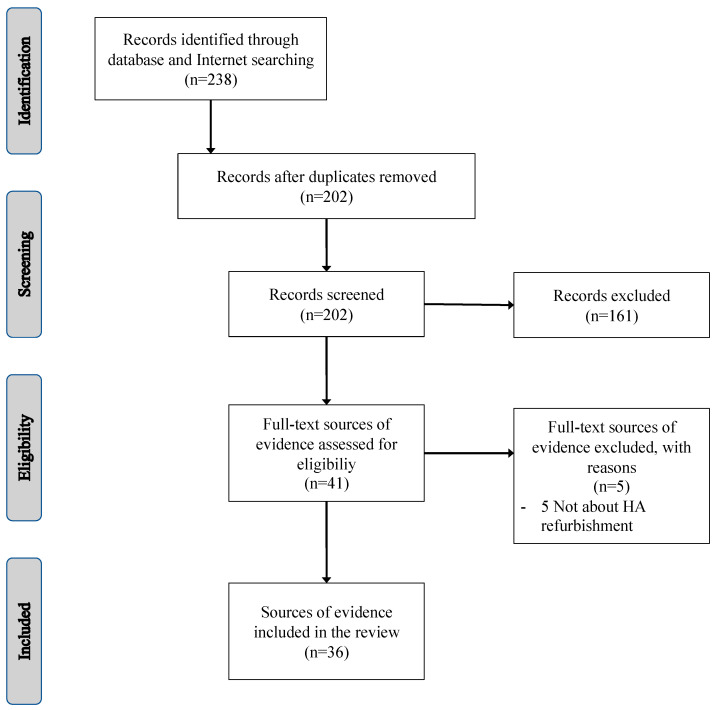
Literature search flow chart.

Following the application of the search strategy, 36 sources of evidence were included in the review. Eleven were articles published in scientific or professional journals. Those 11 articles were used to specifically answer research questions #1 and #2. Twenty-five other sources of evidence were web pages presenting HA refurbishing programs. Those 25 web pages were used to answer research question #3 only. The literature search flow chart is presented in Figure 1. The included sources of evidence can be categorized as quantitative retrospective cross-sectional studies (*n* = 2), qualitative case studies (*n* = 2), narrative or descriptive reviews (*n* = 7), or web pages (*n* = 25). No systematic or scoping review and no experimental or quasi-experimental studies pertaining to HA refurbishing programs were identified. The list of the included sources of evidence related to research questions #1 and #2 is presented in Table A1 (see Appendix A). The list of the 25 HA refurbishing programs that were identified during this review (in relation to research question #3) is presented in Table 2. A summary of the most salient results in relation to each research question is presented below.

### 3.2. Research Question #1: What Are the Advantages and Disadvantages of Refurbished HAs?

Studies were carried out to determine the economic and social benefits that HA-refurbishing programs can have, both for HA recipients and for the government institutions that implant them. Tonning et al. [20] reviewed a system implemented in Norway, where the National Health Insurance provided new and refurbished HAs to their citizens with hearing loss. In that system, a person with hearing loss would be provided new or refurbished HAs, depending on the availability of products at the time of provision. Those HAs were paid for entirely by the government. Repairs of provided HAs were also reimbursed. The authors compared the costs of repairing, refurbishing, and redistributing used HAs with the costs of purchasing new ones. They found that this system resulted in savings at a government level, which means that repair costs were offset by savings made using refurbished HAs instead of only using new devices. In addition, they found that the fact HA repairs were reimbursed by the government resulted in a reduction in the number of people who used faulty HAs or equipment poorly adjusted to their needs, since they would not have to personally pay for replacements and repairs.

#### Impacts of Refurbished HAs for People with Hearing Loss

The identified sources of evidence present experiential knowledge about the impacts, advantages, and disadvantages of refurbished HAs for people with hearing loss, mostly gained while implementing and administrating HA refurbishing services in the community. Unsurprisingly, the most important advantage of refurbished HAs is that they are less expensive than new HAs, which allow more people to access this technology. It is estimated that a refurbished HA can be sold at about 2/3 the price of a new one [21]. Refurbished HAs are generally sold or donated by non-profit organizations to low-income people who cannot afford to buy new devices [22,23,24]. Some authors mentioned that the use of refurbished HAs allowed clients not only to save money, but also to improve communication with coworkers and family and participation in everyday activities [24].

Some disadvantages of refurbished HAs were reported in the literature. Considering the relatively short life cycle of a HA, there is a risk that refurbished HAs become rapidly obsolete due to quick technological advances occurring in the field of hearing amplification [21]. The repair of refurbished HAs may also be an issue, as those technologies are generally repaired using parts collected in other returned HAs or spare parts donated by HA manufacturers [22], and so, the possibility of repairing refurbished HAs depends on the availability of appropriate spare parts. Another disadvantage which is mentioned in the literature is that the characteristics of refurbished HAs may not be appropriate to the needs of all clients with hearing loss. For example, most refurbished HAs are behind-the-ear (BTE) HAs [25]. Used in-the-ear (ITE) HAs can be refurbished, but the process is more expensive, and so, they are usually only used for spare parts [20,22]. Additionally, it may be difficult to find a refurbished HA appropriate for severe or profound hearing losses, as few of these models may be donated [25].

### 3.3. Research Question #2: What Are the Determining Factors for the Success of a HA-Refurbishing Program?

A team of researchers studied the facilitating factors and obstacles to implementing a HA recovery-and-refurbishing system in the UK [21]. According to these authors, to ensure the success of such a system, it is imperative that HA suppliers save money, that an adequate cleaning of the used HAs is made to avoid cross-contamination from one client to another, and that the clients return the devices that were loaned to them when they are not in use anymore. The obstacles preventing the implementation of such a system were the lack of established protocols for the return of HAs, the lack of incentive for clients to return loaned devices, and the rapid technological evolution of HA products, which is related to obsolescence. The authors found that the specific context of the National Health Service in the UK, which was found to focus primarily on the sale and distribution of new HAs, and where no established protocol on the return, disposal, refurbishment, and redistribution of HAs exists, is not favorable to the implementation of a sustainable HA recovery-and-refurbishing system.

**Table 2:** Hearing aid refurbishing programs identified.

Country	Organization Location	Places Where Refurbished HAs Are Distributed	Target Population and Other Eligibility Criteria	Services Available and Price
Australia	EARS, Inc. [26] Templestowe, Victoria.	The Dominican Republic, Fiji, Malawi, Papua New Guinea.	Population of low- and middle-income countries.	Audiological equipment and training, hearing assessment, HAs, assistive listening devices (ALDs), fitting, earmolds.
Canada	Association des personnes avec une déficience de l’audition [15] Québec City, Québec.	Québec, Canada.	Low-income adults and seniors living in Québec, members of the association, not covered by private or public insurance, hearing loss and HA needs assessed by an audiologist.	HAs, ALDs, fitting, earmolds, all free. Membership: 10 CND.
	Dalhousie Hearing Aid Assistance Program [27] Halifax, Nova Scotia.	Nova Scotia, Canada.	Low-income seniors living in Nova Scotia, low income.	Hearing assessment, HAs, fitting, earmolds, follow-up, all free.
	Hearing Solutions [28] Toronto, Ontario.	Peru.	Population of developing countries.	HAs.
	H.E.A.R. Worldwide [29] Ottawa, Ontario.	Canada, developing countries.	Low-income people living in Canada or in a developing country, not covered by private or public insurance.	HAs.
	Robillard Hearing Centres [30] Eastern Ontario.	El Salvador, Peru, Honduras, Palestine, Philippines, China.	Population of developing countries.	HAs.
	Salus Hearing Centre [31] Vaughan, Ontario.	Ontario, Canada.	NA.	HAs.
	Team Canada Healing Hands [32] Montréal, Québec.	Haiti.	Population of Haiti with low income.	Audiological equipment and training, hearing assessment, HAs, fitting, earmolds.
France	Audition Solidarité [33] Yzosse.	France, Dominican Republic, Morocco, Tunisia, Cameroon, Burkina-Faso, Madagascar, Guinea, Vietnam.	France: People with low income, not covered by private or public insurance (Aide Médicale d’État accepted), ENT assessment and HA prescription required. Abroad: underprivileged children only.	France: Hearing assessment, fitting, earmolds, all free. Abroad: Audiological equipment and training, hearing assessment, HAs, fitting, earmolds.
	Idéal Audition [34] Many locations in France.	France, International.	Low-income people living in France or in a developing country.	HAs.
UK	DeafKidz International [35] Brighton.	UK, Gambia, India, Jordan, Malawi, Pakistan, Rwanda, Sierra Leone, South Africa, Zambia, Zimbabwe.	Deaf children, young people, and adults living in UK or in a developing country, low income.	Audiological equipment and training, hearing assessment, HAs, fitting, earmolds, Sign Language training.
	Hearing Care Centre [36] Suffolk, Norfolk.	International.	Population of developing countries.	HAs.
USA	Hearing and Speech Foundation [37] Maryville, Tennessee.	USA, Jamaica.	Residents of East Tennessee (children and adults), gross household income does not exceed 100% above the federal poverty level.	Hearing assessment, HAs, accessories, ALDs, fitting, earmolds. HAs are free. Sliding scale fee system for services based on household income, from 145 to 350 USD.
	Rotary [38] Many locations in USA.	Argentina, Philippines, Dominican Republic	Low-income population in developing countries.	Supports projects to provide affordable HAs and services in underserved areas.
	Olive Osmond Hearing Fund [39] St. George, Utah.	USA.	Low-income people (children and adults) who cannot afford HAs and have no resources available to obtain amplification devices.	HAs, other devices, or audiology services on a case-by-case basis.
	Grace Hearing Center [40] Tucson, Arizona.	USA.	Residents of the greater Tucson area (children and adults), household income does not exceed 250% above the federal poverty level, willingness to do community volunteering.	Hearing assessment, HAs, accessories, fitting, earmolds. Sliding scale fee system for HAs (including services) based on household income, from 80 to 450 USD, plus a designated amount of volunteer hours in the community.
	GiveHear [41] Fort Wayne, Indiana.	USA.	Residents of northeast Indiana (children and adults), household income does not exceed 250% above the federal poverty level, willingness to do community volunteering.	Hearing assessment, HAs, accessories, fitting, earmolds. Sliding scale fee system for HAs and services based on household income, plus patients are asked to do volunteer hours in the community.
	Hearing Charities of America [42] Kansas City, Missouri.	USA.	Residents of the USA (children and adults), hearing loss diagnosed and HA recommendation by an audiologist, low income without health insurance or coverage for HAs.	Hearing assessment, HAs.
	Pacific Neuroscience Institute Foundation [43] California.	USA.	Hearing loss diagnosed by an audiologist, low income without health insurance or coverage for HAs.	HAs.
	Southern Arizona hearing aid bank [44] Tucson, Arizona.	USA.	Low-income adults unable to afford hearing care.	Services and HAs, 95 USD.
	The UWSHC Hearing Aid Recycling Program [45] Madison, Wisconsin.	USA.	Low-income residents of Dane County and surrounding area. People with significant hearing loss in both ears are prioritized. Reasonable benefit from the HAs expected by the audiologist.	Hearing assessment, HAs, fitting, earmolds, initial follow-up, all free. Other audiology services, programming, and repair provided on a case-by-case basis.
	Hearing the Call [46] Fort Wayne, Indiana.	USA, Jordan, Palestine, Mexico, Mozambique, Zambia, South Africa, India, Brazil, Guatemala, Ecuador.	Population of developing countries. Residents of the USA (children and adults), household income does not exceed 250% above the federal poverty level, willingness to participate in community volunteering.	Hearing assessment, HAs, accessories, fitting, earmolds. Sliding scale fee system for HAs and services based on household income, plus patients are asked to perform volunteer hours in the community.
	Hope for Hearing [47] Ann Arbor, Michigan.	USA.	Low-income residents of Washtenaw County.	HAs.
	Tulsa Speech and Hearing Association [48] Tulsa, Oklahoma.	USA.	Low-income residents of Oklahoma. Hearing test by an audiologist (no older than 6 months), 30 dB hearing loss in the better ear.	1 HA, 75 USD.
International	Lions Clubs International [49] Many locations in the world.	International, Local.	NA.	HAs.

Note. Specific information about each identified HA refurbishing program was added when available online. ENT: ear, nose, and throat doctor. NA: not available.

Another determining factor that emerged from Tonning et al.’s [20] study was the importance of the cooperation of hearing healthcare professionals and their attitudes towards the use of refurbished Has. Particularly, it appears crucial that professionals actively participate in the program by making clients aware of the importance of the collection of HAs that are no longer in use.

When a used HA is returned to the distributor, it is not necessarily easy for the professional to know when it is advantageous to refurbish it, or to send it for recycling or disposal. To help professionals make this choice, Rudi et al. [50] developed a software which takes many factors into consideration (i.e., the age of the technical aid, its life cycle, price, cost of disposal, expected time to refurbish the aid, etc.), and which makes it possible to support the decision to refurbish a technical aid or not. This software was created for the Norwegian National Health Insurance program, which, at that time, lent out technical aids, from wheelchairs to HAs, or even adapted cars. The use of the software was tested in various lending centers in Norway and professionals found plenty of advantages in its use. They mentioned that it helped them to consider several factors in their decisions that they previously overlooked, and that it changed the way they determined what aid could be refurbished. As many factors were considered, therefore allowing professionals to obtain an objective proof of the possible advantages, they noticed that they would refurbish much more aids than before, avoiding ending their life in the landfill. The software made it possible to highlight the economic advantages that the refurbishing of technical aids can generate, while making professionals aware of its possible benefits, which made the decision-making process easier and more objective.

Finally, authors mentioned that it is not sufficient to simply distribute refurbished HAs to people in need freely or at a low cost. Accessible and affordable follow-up services, repairs, and batteries are essential for the sustainable use of refurbished HAs [22,23,51,52].

### 3.4. Research Question #3: What Are the Existing Programs Which Distribute Refurbished HAs to People with Hearing Loss around the World?

The 25 HA refurbishing programs that were identified during this review were all based in developed countries. They distributed refurbished HAs (mostly BTE HAs) in developing countries (*n* = 6), in local communities (*n* = 12), or both (*n* = 7). All programs targeted low-income patients without other resources available to obtain amplification devices. This implies that patients must qualify to receive refurbished HAs from most programs by filling an application form and sending personal data such as tax slips, an audiogram, and a HA recommendation. Little information is available online about the other characteristics of identified HA refurbishing programs (e.g., specific eligibility criteria, number of HAs allotted per patient, if other hearing assistance technologies are refurbished by the organization, and price). Some programs were found to have minimum audiometric eligibility criteria [48], require that the patient’s hearing loss was diagnosed and documented by a licensed audiologist [15,42,43,45,48], or only accepted patients living in a specific geographic area [15,27,37,40,41,45,47,48]. One organization mentioned that only one refurbished HA was distributed to each eligible patient [48]. Another organization also refurbished assistive-listening devices [15,26,37]. When mentioned, the cost of one refurbished HA for eligible patients was variable. Some programs used a fixed rate of 0 USD [15,27,45] or less than 100 USD [44,48], while other programs used a sliding scale fee system based on household income with a maximum price of less than 500 USD per HA [37,40]. One program required the patient to pay a one-year membership of 10 CND to the association running the program [15], and three others asked the patient to perform volunteer work in the community in exchange for the HA [40,41,46].

## 4. Discussion

This exploratory scoping review focused on describing the impacts of refurbished HAs for people with hearing loss, and on identifying existing HA refurbishing programs around the world. According to the included sources of evidence, refurbished HAs may provide monetary savings to citizens with hearing loss and, more globally, to governmental agencies which distribute refurbished HAs to their population. It may also improve communication and social participation for individuals with hearing loss in their everyday life. However, cross-contamination, quick obsolescence, and repairing issues with refurbished HAs could represent potential problems for users of those devices. Determining factors for the success and a sustainable use of refurbished HAs were also identified. For a HA refurbishing program to be successful, it is necessary that hearing healthcare professionals collaborate with it by proposing the option to their patients in need, by offering accessible and affordable follow-up services, repairs, and batteries, and by informing patients of the possibility of returning their old HAs for refurbishment. Additionally, a clear protocol on the return, disposal, refurbishment, and redistribution of HAs must be established. Therefore, it appears that individuals with hearing loss may benefit from refurbished HAs even if this option might present some limitations; however, the use of those devices should be a part of a more global intervention program in order to be sustainable.

In this review, 25 HA refurbishing programs were identified. Those programs were all located in developed countries. This is not surprising, as HAs are more common and HA market penetration is greater in those countries. However, the fact that the majority of the identified programs distribute refurbished HAs locally, inside developed countries such as the USA, the UK, Canada, and France, where public healthcare systems and private health insurance plans are in place, was unexpected. This may be explained by an incomplete coverage or reimbursement of HAs by those instances in developed countries and by the high cost of HAs, leaving many low-income people without any other means for obtaining HAs than refurbishing programs. For example, in the Province of Québec, Canada, adults with hearing loss must present with a pure-tone average greater or equal to 35 dB HL in their best ear to qualify for the coverage of one HA by the Québec Health Insurance Board; the second HA is only covered for those active in the labor market [53]. This is an illustration that refurbished HAs may be a valuable way to meet the needs of low-income people with hearing loss not only in developing countries but also in developed countries.

Available information about specific characteristics of identified HA refurbishing programs showed that all those programs targeted low-income people and that some of them required the patient to meet some eligibility criteria before entering the program. Then, refurbished HAs are not accessible to everyone. People with hearing loss who would like to obtain refurbished HAs should expect to be questioned by the organization running the program about their income, living situation, and other personal data. They should also know that refurbished HAs are not necessarily free, that a monetary or even a volunteering contribution might be requested from them to gain access to those aids.

Although some HA-refurbishing programs were identified around the world, those initiatives remain generally unknown from the public. Is it due to a poor visibility of HA-refurbishing programs in the public space, a lack of resources in community organizations operating those programs, an insufficient public awareness about hearing loss and HAs, or other reasons? No information was found in the literature about this specific question. It would be important to explore this in future research to gain a better understanding of the situation and help HA-refurbishing programs to reach a larger audience.

In addition, the benefits of HA-refurbishing programs for individuals were little documented so far in the scientific literature. In this scoping review, we did not assess the scientific quality of the included sources of evidence, but if we look at the research designs used in included studies, it suggests that the scientific quality of those studies is low. In fact, most of the 11 scientific articles included in this review were narrative or descriptive reviews, and no experimental or quasi-experimental studies were found. Therefore, the knowledge base available about refurbished HAs appears very limited. More research is needed in this field to better describe and understand the impacts of the use of refurbished HAs for patients and the factors associated with the success of this intervention.

This review focused on HA technologies, but refurbishment was used for many years with other assistive technologies (AT) in different health sectors, such as occupational therapy and optometry. A quick look at the literature pertaining to wheelchairs, mobility aids, and eyeglasses refurbishment shows that the offer of those refurbished AT, as for refurbished HAs, allow for meeting the needs of people and may provide monetary savings to governmental agencies and to low-income individuals who have disabilities [54,55,56,57]. Still, the effectiveness of AT-refurbishing programs depends on the awareness and participation of healthcare providers and patients. This supports the results of the present scoping review, as identified impacts and determining factors related to HA refurbishing appear similar to those reported for other refurbished AT.

### Study Limitations

As mentioned previously, few scientific data are available about HA refurbishment. In this scoping review, only 11 articles related to research questions were included. Those articles presented results exclusively from quantitative retrospective cross-sectional studies, qualitative case studies, or narrative/descriptive reviews, which are research designs of limited scientific quality. Moreover, information about specific characteristics of many identified HA-refurbishing programs was not accessible on consulted websites. In this context, the results of this review should be interpreted with caution. More research of better quality is needed about HA refurbishing programs. Professionals who might be interested in participating in such initiatives or patients who would like to obtain refurbished HAs are encouraged to contact a local organization for further details.

## 5. Conclusions

The use of refurbished HAs appears to be a valuable option for low-income people living with hearing loss. It may improve communication and social participation for those individuals and provide monetary savings to them and to governmental agencies which distribute HAs to their population. However, cross-contamination, quick obsolescence, and repairing issues could represent potential problems of refurbished HAs. When implementing a HA-refurbishing program, it is important not only to plan how the HAs will be refurbished and redistributed, but also to determine a clear protocol for HA return and to explore the possibilities of offering accessible and affordable follow-up services, repairs, and batteries. Including the refurbished HAs in a more global intervention program should ensure its sustainability. Awareness and participation of hearing healthcare professionals and of individuals with hearing loss are also critical for the success of such a program. More research is needed on refurbished HAs. Future research should focus on describing and understanding the impacts of the use of refurbished HAs for individuals and the factors associated with the success of this intervention.

## Figures and Tables

**Table 1:** Keywords and combinations used for databases’ consultations.

Keywords Related to Technology		Keywords Related to Refurbishment
hearing aid OR hearing device OR hearing technology OR hearing assistance technology OR hearing assistive device OR hearing assistive aid OR assistive listening device OR hearing instrument	AND	recycl* OR refurbish* OR valoriz* OR valoris*

## Data Availability

Not applicable.

## Appendix A

**Table A1:** List of included sources of evidence related to research questions #1 and #2.

Authors (Year)	Country	Design	Funding Source	Participants	Main Results
Carkeet et al. [22]	Australia, Dominican Republic	Review	Not mentioned	n/a	Present a model developed by Ears Inc., which was implemented in Dominican Republic in partnership with Centro Cristiano de Servicios Medicos. Donated HAs are used for patients who cannot afford to purchase a new one. These BTE HAs are checked and cleaned by the technician prior to supply. Patients are asked to pay a small fee for the fitting of these devices which covers the cost of the clinician’s time. Only in very rare cases are donated HAs provided at less than the cost of the appointment. Donated ITE HAs are only used for spare parts. Donated HAs are typically repaired by searching for pieces on other donated products or from parts donated from manufacturers. Follow-up services, repairs, local ear mold manufacture, and batteries are essential for sustainable use of HAs.
Cole et al. [21]	UK	Qualitative research, semi-structured interviews	University of Surrey, Edinburgh Napier University	8 audiologists, 6 National Health Service (NHS) representatives, 6 HA manufacturer representatives	Topic = Reverse exchange of HAs in the UK. Refurbishment and reuse of HAs exist in the UK. Refurbished HAs are cheaper than new HAs, about 2/3 the price of a new HA. There is a risk for cross-contamination; a rigorous and effective refurbishment is needed to control this risk. HAs must be returned by the client when no longer needed.
Manchaiah et al. [23]	India	Review	Not mentioned	n/a	Audiology India (AI) has received free refurbished HAs from various sources, but mainly from the Hearing Aids Recycle Program (HARP) of Lions Club in the United Kingdom. These HAs are provided free of charge to individuals diagnosed with hearing loss during community services. There is a close involvement of the people from local organizations and support groups to allow this program to be successful.
Mbibeh et al. [58]	Cameroon	Qualitative research, interviews, focus groups	Canadian Institutes of Health Research (CIHR), Cameroon Baptist Convention Health Services	Practitioners, duty bearers, service providers, service users and persons with hearing impairments (sample size not mentioned)	There is a severe shortage of ear and hearing services, limited accessibility to medical products and health technologies, no training avenues locally available and no clear strategy to identify and address components and gaps in Cameroon. The study recommends an urgent need for the design of a strategic plan to address the gaps which should include strong representation from the deaf and hard-of-hearing communities. At the time this study was conducted, Sound Seekers from UK were active in the country to help with that situation. Generally, Zambian sources refurbish and donate HAs to Mbingo Baptist Hospital (MBH), where people can go to obtain some hearing healthcare services. Additionally, new HAs and batteries are available.
McBride [51]	USA, Malawi	Review	Not mentioned	n/a	Arizona State University (ASU) Hearing for Humanity (HFH) receives donations of new and used devices from manufacturers, audiologists, and patients of the ASU Speech and Hearing Clinic. With fundraising efforts, HFH is also able to purchase new HAs through the International Humanitarian Hearing Aid Purchasing Program (IHHAPP). To fit HAs responsibly in a developing country, the author suggests to: (1) only fit people who can benefit from a HA, (2) fit HAs according to evidence-based practices, (3) ensure that follow-up care is available and provided either locally or, at a minimum, through annual visits by an outside team, (4) offer education and training for patients as well as teachers and parents who help taking care for the HA. The program is operated in partnership with Sound Seekers and EARS Inc.
Parmar [25]	UK	Review	Not mentioned	n/a	Sound Seekers operate in Malawi, Zambia, Gambia, Sierra Leone, and Cameroon. HAs are collected in the UK, and are then shipped to Zambia to be refurbished. Some challenges encountered are presented and discussed. (1) Used HAs are often donated without key components such as the HA ear hook or battery compartment. Such a HA proves unusable unless spare parts can be found or ordered. (2) Digital HAs from the NHS audiology departments require specific programming accessories and software which may result in a delay in refurbishment and subsequent fitting. (3) A significantly lower number of power HAs are donated which may limit the capacity of the program to meet the needs of patients with a severe or profound hearing loss. (4) Only behind-the-ear (BTE) HAs are refurbished; in-the-ear (ITE) HAs are only used for spare parts.
Parmar et al. [52]	UK, Malawi	Descriptive, Cross sectional, Retrospective	Not mentioned	2299 patients, 1521 adults (age: M = 45.8, SD = 19.2) and 778 children (age: M = 7.7, SD = 5.2)	Description of an audiology clinic in Malawi, developed with the help of Sound Seekers, now Deaf Kidz International. Refurbished HAs come from a Zambia lab. Overall, in the 2016–2017 period, the percentage of patients diagnosed with a hearing loss in this clinic was 61.6% for adults and 41.7% for children. Uptake of HAs was 28.3% for adults and 15.4% for children identified with a hearing loss. Reasons for this HA uptake rate are: (1) Few donated HAs for profound hearing loss, (2) Practical barriers to uptake exist (ex. indirect costs of fitting, such as food, travel and accommodation expenses for follow-up, HA after care and battery costs), (3) General lack of awareness about hearing loss, cultural negative opinions and stigma associated with hearing loss and HAs, negative social representation of HAs. Authors made recommendations about the operation of a refurbished HA program in a developing country: (1) Use of low-cost hearing technologies, (2) Favor models of community-delivered hearing care, (3) Improve training of personnel.
Pither [59]	Australia	Review	Not mentioned	n/a	Presentation of EARS, Inc. which trains locals with the help of volunteers. They had mission in Vanuatu, Vietnam, Philippines, Cambodia, India, New Guinea, Ukraine, Tajikistan, Ethiopia, Botswana, Swaziland, Zimbabwe, Malawi, and Dominican Republic. They use new low-cost, used, and refurbished HAs.
Rudi et al. [50]	Norway	Review, qualitative research	Not mentioned	35 employees and administrators of the Norwegian National Insurance Administration (NNIA)	Presents a review and a model about product recovery decision making applicable for different types of technical aids (TA), including HAs. The model presented is used at the NNIA, which refurbish technical aids. Comments from NNIA employees and administrators about their model are also presented. The model facilitates decision making about whether to refurbish a TA or not. It increases the amount of TA refurbished and reduces the number of units sent unnecessarily to landfills.
Tonning et al. [20]	Norway	Descriptive, Cross Sectional, Retrospective	Not mentioned	n/a	Presentation of Norway’s National Health Insurance (NHI) HA refurbishment program. Authors analyzed numbers and proportions of returned HAs that have been refurbished/reused and the associated costs. Reuse of HAs can help to cover costs of repairing other HAs. The allocation of 1 reused HA (instead of buying a new one) can save sufficient money to repair approx. 6 broken HAs, and so, refurbishment helps to sustain free repair of HAs by government. However, financial savings from reusing ITE are lower than BTE. Attitudes of the audiology personnel regarding reuse is very important and it must be beneficial for the patient. Collecting used HAs is necessary; in that regard, a system must be put in place. The state of the HA is important when deciding to reuse it or not (discard if HA too old or too broken). Around 10% of returned HAs were functioning perfectly.
Whitley [24]	USA	Review	Not mentioned	n/a	Presents the Hearing Charities of America (HCOA) Hearing Aid Project, and examples of benefits experienced by individuals. A few short testimonies of patients are presented. Refurbished HAs brought money savings to patients and a better communication with coworkers and family.
